# Risk factors for relapse among patients with alcohol dependence syndrome

**DOI:** 10.6026/973206300211034

**Published:** 2025-05-31

**Authors:** Nalini S., Vijayasamundeeswari P., Sara Sapharina G.J., Poongodi R.

**Affiliations:** 1 Department of Psychiatric Nursing, Sri Ramachandra Faculty of Nursing, Sri Ramachandra Institute of Higher Education and Research (DU), Porur, Chennai-600116, India

**Keywords:** Risk factors, relapse, alcohol dependence syndrome

## Abstract

Alcohol dependence poses significant challenges globally, with relapse prevention being a crucial aspect of treatment. This study, conducted among patients with alcohol dependence syndrome in psychiatric and outpatient departments at a tertiary hospital, examined risk factors for relapse. The findings highlighted that family dynamics significantly influence relapse risk, while selected clinical variables showed no significant association. Notably, craving for alcohol, pleasurable events, family conflict, financial problems and loss of loved ones emerged as major risk factors for relapse or lapse. The majority of participants were found to have a moderate risk for relapse. Thus, the importance of tailored interventions by healthcare providers to prevent relapse and enhance patient quality of life is relevant.

## Background:

Alcohol dependence is a serious social, psychological and medical problem in many parts of the world. It has been showing a rising trend all over the world including India, perhaps as a result of newer and greater stresses related to rapid changes in lifestyles. Each year abuse of alcohol has an enormous toll in death, decline in productivity, more crime and accidents and also increases the expenditure in rehabilitation [1]. It is a chronic as well as a relapsing disorder. Even with improvement of multiple domains in life with treatment, the relapse risk continues to be high after treatment. A relapse happens when a person stops maintaining his or her goal of reducing or avoiding use of alcohol or other drugs and returns to previous levels of use [2]. Alcohol abuse is a disorder associated with an unhealthy pattern of alcohol consumption and it causes social problems for the individual. These problems include lack of responsibility in home, workplace, school and even legal problems for the individual [3]. The World Health Organization estimates the presence of 140 million alcoholics worldwide. Studies suggest that patients who received treatment within 30 days of completing detoxification were ten times less likely to relapse, while those completing detoxification alone are getting relapsed at the rate of 65%-80% [4]. Relapse of alcohol dependence is a sensitive social issue, may be related to various factors related to client, family and society causing never-ending loss to the nation with regard to the health and wealth of every citizen affected [5]. The main reason for relapse is concurrent depression or anxiety symptoms, low education and lack of motivation for abstinence [6]. It significantly impairs the prognosis of treatment outcomes. Craving was noted as most common cause for relapse in alcohol dependent patients [7]. Relapse is common among alcohol dependence and it seriously complicates treatment. It is estimated that 60% to 75% of those who complete treatment programme, drink again within first 90 days. As a result of a successful initial change, the person experiences perceived control while remaining abstinent [8]. This feeling of perceived control continues until the person encounters a high-risk situation involving negative emotional states, interpersonal conflict, or social pressure [9]. It is one of the hallmarks of alcohol dependence and constitutes therapeutic challenge throughout the life of alcohol dependence. There is continuing and growing concern among clinicians about the high rate of relapse among alcohol dependence patients and increasingly adverse consequences of continuing disease. Approximately 90% of alcoholics are likely to experience at least one relapse over the four-year period following treatment [10]. Relapse prevention is a major challenge in the treatment of alcohol dependence. About 50% of detoxified alcohol dependent individuals relapse within 3 months. Identification of the factors leading to relapse in alcoholics may serve as an important step in suggesting appropriate interventions and continuing care regimens for those most at risk [11]. It is a cognitive-behavioural strategy aimed at recognizing and managing high-risk situations to prevent relapse while supporting individuals in sustaining positive behavioural changes [12]. Relapse is a serious problem in the treatment of alcoholism. So therefore, relapses must be dealt with and seen as a sign to the alcoholic and need serious work to be done in treatment and recovery of alcoholism [13]. This study, conducted among patients with alcohol dependence syndrome in psychiatric and outpatient departments at a tertiary hospital, examined risk factors for relapse.

## Methodology:

## Research design and sample:

A descriptive research design was adopted for this study. The study was conducted in psychiatric outpatient and inpatient departments of Sri Ramachandra hospital (SRH). The sample for the study includes 60 patients with risk for relapse attending psychiatric outpatient and inpatient department of Sri Ramachandra Hospital, who are available during data collection and who filled the inclusion criteria. The sample for the study includes 60 patients with alcohol dependence syndrome and they were selected using non probability convenience sampling technique. The inclusion criteria for sampling selection were patients with alcohol dependence syndrome who are able to understand Tamil or English and willing to participate in the study. Patients with alcohol dependence syndrome who have with chronic physical or mental illness, cognitive impairment and who need more intensive treatment were excluded.

## Ethical considerations:

The study was approved by Institutional Ethics Committee (IEC) (Project No.CSP 22/ JAN/104/17), dated November 2, 2022 and informed consent was obtained from the study participants.

## Data collection:

Rapport was maintained and explained the purpose of the study to the patients. The researchers interviewed 60 study subjects and background variables of patient such as age, gender, type of family and residence, education, occupation, monthly income, marital status, religion and the clinical variables of patient such as age of initiation of alcohol intake, amount of consuming alcohol/day, duration of consuming alcohol, family history of alcohol use, no. of previous relapse, history of de addiction treatment and the risk for relapse was assessed by the Alcohol Relapse Risk Scale (ARRS) Scale [14]. Comfort and their privacy were taken into considerations. Confidentiality was maintained throughout the study. The time taken for the data collection from each participant was about 20 mints using English and Tamil language. After the completion of the questionnaire, each patient was thanked individually. The data obtained for the present study were analyzed using descriptive and inferential statistics.

## Discussion:

The present study revealed that the majority of the patients 45 (75.00%) initiated alcohol intake in the age group of 20-30 years. Considering the amount of alcohol consuming / day 25 (41.67%) of them were consuming -90ml of alcohol per day. In viewing the duration of consuming alcohol 44 (73.33%) of them were 6-10 years. Regarding the family history of alcohol use 43 (71.67%) of them doesn't have the family history of alcohol use. Considering the no. of previous relapse 21 (35.59%) of them had 1 previous history of relapse. In viewing the history of previous de- addiction treatment 45 (75.00%) of had a history of previous de - addiction treatment. The study's findings offer important insights into the risk stratification of patients regarding relapse. According to the data, a vast majority-83.33% (50 patients)-were classified as having a moderate risk of relapse, indicating that while these individuals are not at the highest level of risk, they still require careful monitoring and potentially tailored interventions to prevent relapse [15]. The 10% (6 patients) who fall into the high-risk category are particularly concerning, as these individuals may require more intensive follow-up, support, or possibly adjustments to their treatment plans to reduce their likelihood of relapse. On the other end of the spectrum, only 6.67% (4 patients) were considered at low risk, suggesting that only a small portion of the cohort may not need extensive post-treatment monitoring [16]. The mean score was 59.33 with standard deviation of 6.24 for risk factors for relapse among patient with alcohol dependence syndrome. The findings are supported by a descriptive study conducted on Risk of relapse in clients with alcohol dependence syndrome in a tertiary care hospital. This study highlighted that 63 (67.7%) were in the low-risk group for getting relapse, 26 (27.7%) were in the moderate-risk group and 5 (5.3%) were in the high-risk group [17].

Table 1 reveals the association between level of risk factors for relapse with selected background variables among patients with alcohol dependence syndrome (N=60). The study found a significant association between family type and relapse risk in patients with Alcohol Dependence Syndrome (ADS) at p-0.01, highlighting the influence of family structure on recovery outcomes [18]. However, no significant associations were observed between relapse risk and other socio-demographic factors such as age, education, occupation, monthly income, marital status, or residence at the same significance level. This suggests that while family dynamics play a crucial role in relapse, other demographic variables may not have a direct impact. Therefore, interventions focusing on family support systems could be more effective in preventing relapse among ADS patients. Further research is needed to explore these relationships in diverse populations [19]. The study found no significant association between relapse risk and clinical variables such as age at initiation of alcohol intake, amount and duration of consumption, family history of alcohol use, number of previous relapses, and history of prior de-addiction treatment among patients with Alcohol Dependence Syndrome (ADS). This indicates that these clinical factors may not be primary predictors of relapse in this population. Instead, psychosocial elements-like coping behaviors, stress levels, and social support-may play a more pivotal role in influencing relapse risk. Therefore, focusing on enhancing coping strategies and providing robust psychosocial support could be more effective in preventing relapse among ADS patients. These findings underscore the importance of comprehensive treatment approaches that address psychological and social dimensions of recovery [20].

## Conclusion:

Majority of the study participants had moderate risk for relapse. The findings are useful in guiding healthcare providers to develop essential interventions to prevent relapse. This study can provide basis for planning effective interventional strategies for relapse prevention which ultimately improves the quality of life of patient.

## Figures and Tables

**Table 1:** Association between level of risk factors for relapse with selected background variables among patients with alcohol dependence syndrome (N=60)

**Background variables**		**Level of Risk factors for Relapse**					**Chi -square**	**P value**
	**Low risk**		**Moderate Risk**		**High Risk**			
	**n**	**%**	**n**	**%**	**n**	**%**		
Age in Years							4.442	0.368
31 - 40	0	0	12	20	2	3.33		(NS)
41 - 50	3	5	15	25	1	1.66		
51 - 60	1	1.66	23	38.33	3	5		
Education							10.132	0.256
No formal education	0	0	14	23.33	3	5		(NS)
Primary education	2	3.33	14	23.33	0	0		
Secondary education	2	3.33	7	11.66	1	1.66		
Higher education	0	0	6	10	0	0		
Graduate	0	0	9	15	2	3.33		
Occupation							4.756	0.575
Coolie worker	2	3.33	16	26.66	4	6.66		(NS)
Employed	2	3.33	19	31.66	1	1.66		
Retired	0	0	6	10	0	0		
Unemployed	0	0	9	13	1	1.66		
Monthly Income (In Rupees)							4.542	0.338
Rs.>10000	1	1.66	27	45	4	6.66		(NS)
Rs.10001-20000	2	3.33	8	13.33	0	0		
Rs.-20,001	1	1.66	15	25	2	3.33		
Marital Status							6.05	0.195
Married	1	1.66	31	51,66	6	10		(NS)
Unmarried	2	3.33	13	21.66	0	0		
Separated/Divorced	1	1.66	6	10	0	0		
Type Of Family							9.888	0.007
Nuclear family	4	6.66	21	35	0	0		**(S)
Joint family	0	0	29	48.33	6	10		
Residence								0.411
Rural	3	5	29	48.33	5	8.33		(NS)
Urban	1	1.66	21	35	1	1.66	1.777	
**p-.01, S-Significant,
NS-Non Significant
